# Attending Families by the Year

**Published:** 1851-01

**Authors:** 


### ARTICLE XIV.

### *Attending Families by the Year.*

The Plan of attending famlies by the year is dictated by a trading mercenary spirit, unworthy of members of a dignified profession. It has too much the air of pelf about it, and should at once be abandoned. It may suit the dealer in matches, the butcher, or the iceman, but is clearly out of place among physicians. We are glad to hear that notwithstanding the efforts of some members to have it recognised by the Medical Society, that respectable body unhesitatingly condemned it.—*St. Louis Probe.*

